# Supplementary material for: Artificial light during the polar night disrupts Arctic fish and zooplankton behaviour down to 200 m depth
Source: Commun Biol. 2020 Mar 5;3:102. doi: 10.1038/s42003-020-0807-6 (PMC7058619; doi:10.1038/s42003-020-0807-6)
Supplement: Supplementary file 1 — Reporting Summary [file 42003_2020_807_MOESM1_ESM.pdf]

nature research

Corresponding author(s): Jørgen Berge

Last updated by author(s): 19 Sept 2019

Reporting Summary

Nature Research wishes to improve the reproducibility of the work that we publish. This form provides structure for consistency and transparency in reporting. For further information on Nature Research policies, see Authors & Referees and the Editorial Policy Checklist.

Statistics

For all statistical analyses, confirm that the following items are present in the figure legend, table legend, main text, or Methods section.

n/a	Confirmed	
☐	☒	The exact sample size (*n*) for each experimental group/condition, given as a discrete number and unit of measurement
☒	☐	A statement on whether measurements were taken from distinct samples or whether the same sample was measured repeatedly
☒	☐	The statistical test(s) used AND whether they are one- or two-sided<br>*Only common tests should be described solely by name; describe more complex techniques in the Methods section.*
☒	☐	A description of all covariates tested
☒	☐	A description of any assumptions or corrections, such as tests of normality and adjustment for multiple comparisons
☒	☐	A full description of the statistical parameters including central tendency (e.g. means) or other basic estimates (e.g. regression coefficient) AND variation (e.g. standard deviation) or associated estimates of uncertainty (e.g. confidence intervals)
☒	☐	For null hypothesis testing, the test statistic (e.g. *F*, *t*, *r*) with confidence intervals, effect sizes, degrees of freedom and *P* value noted<br>*Give P values as exact values whenever suitable.*
☒	☐	For Bayesian analysis, information on the choice of priors and Markov chain Monte Carlo settings
☐	☒	For hierarchical and complex designs, identification of the appropriate level for tests and full reporting of outcomes
☒	☐	Estimates of effect sizes (e.g. Cohen's *d*, Pearson's *r*), indicating how they were calculated

Our web collection on statistics for biologists contains articles on many of the points above.

Software and code

Policy information about availability of computer code

Data collection	All acoustic data were treated and analysed in the Echoview software
Data analysis	No custom code or mathematical algorithm was deemed central to the conclusions.

For manuscripts utilizing custom algorithms or software that are central to the research but not yet described in published literature, software must be made available to editors/reviewers. We strongly encourage code deposition in a community repository (e.g. GitHub). See the Nature Research guidelines for submitting code & software for further information.

Data

Policy information about availability of data

All manuscripts must include a data availability statement. This statement should provide the following information, where applicable:

- Accession codes, unique identifiers, or web links for publicly available datasets
- A list of figures that have associated raw data
- A description of any restrictions on data availability

All acoustic and light measurement data are available on the Polar Data Catalogue (https://www.polardata.ca/) under access code CCIN 13104.

Field-specific reporting

Please select the one below that is the best fit for your research. If you are not sure, read the appropriate sections before making your selection.

☐ Life sciences ☐ Behavioural & social sciences ☒ Ecological, evolutionary & environmental sciences

For a reference copy of the document with all sections, see nature.com/documents/nr-reporting-summary-flat.pdf

Ecological, evolutionary & environmental sciences study design

All studies must disclose on these points even when the disclosure is negative.

Study description	A field study where we used acoustics, trawls and light measurements to investigate the impact on vertical placement and distribution of pelagic biomass
Research sample	The samples were first of all acoustic surveys backed up with trawls / zooplankton nets for a groundtruthing of which species were detected on the acoustics
Sampling strategy	not appliccable
Data collection	Data were recorded using ship-bourne acoustic instruments
Timing and spatial scale	The field experiments were carried out over the course of 10 days in January 2018
Data exclusions	No data have been excluded from the analyses
Reproducibility	acoustic survey were carried out over a given time window to avoid any point measurements
Randomization	not appliccable
Blinding	not appliccable

Did the study involve field work? ☒ Yes ☐ No

Field work, collection and transport

Field conditions	The study was carried out in the polar night (continuous darkness) at sea, with water temperatures close to zero and air temperatures between -5 and -25C
Location	One location outside Tromsø (70N) and two locations around the archipelago of Svalbard (76 and 77N)
Access and import/export	The only way to access these areas are by using research vessels. In our case we used the RV Helmer Hanssen. All neccassary approvals and licences were provided by national and local governments
Disturbance	The study was aimed at how light pollution actually disturb pelagic organisms. We have documented its impact, and our results can be used to minimize disturbance in the future

Reporting for specific materials, systems and methods

We require information from authors about some types of materials, experimental systems and methods used in many studies. Here, indicate whether each material, system or method listed is relevant to your study. If you are not sure if a list item applies to your research, read the appropriate section before selecting a response.

Materials & experimental systems

n/a	Involved in the study	
☒	☐	Antibodies
☒	☐	Eukaryotic cell lines
☒	☐	Palaeontology
☐	☒	Animals and other organisms
☒	☐	Human research participants
☒	☐	Clinical data

Methods

n/a	Involved in the study	
☒	☐	ChIP-seq
☒	☐	Flow cytometry
☒	☐	MRI-based neuroimaging

Animals and other organisms

Policy information about studies involving animals; ARRIVE guidelines recommended for reporting animal research

Laboratory animals	No lab experiments were performed
Wild animals	Wild animals were either studied using acoustics (non-invasive) or by pelagic / bottom trawling. All nessaccary licences and approvals were secured to carry out the trawling, which were always kept to an absolute minimum. The purpose of the trawling were only to provide a groundthruthing of the pelo9agic communities during the light and acoustic experiments

Field-collected samples	Three field experiments were carried out, all over a period of up to 6 hours. Sampling of organisms (trawling) was limited to 20 minutes.
Ethics oversight	No ethical approval was required

Note that full information on the approval of the study protocol must also be provided in the manuscript.
